# Interlaboratory Comparison on High-Temperature Superconductor Critical-Current Measurements

**DOI:** 10.6028/jres.102.004

**Published:** 1997

**Authors:** J. A. Wiejaczka, L. F. Goodrich

**Affiliations:** National Institute of Standards and Technology, Boulder, CO 80303

**Keywords:** critical current, degradation, high temperature superconductors, interlaboratory comparison, repeatability, standard method, variability

## Abstract

An extensive interlaboratory comparison was conducted on high temperature superconductor (HTS) critical-current measurements. This study was part of an international cooperative effort through the Versailles Project on Advanced Materials and Standards (VAMAS). The study involved six U.S. laboratories that are recognized leaders in the field of HTS. This paper includes the complete results from this comparison of critical-current measurements on Ag-sheathed Bi_2_Sr_2_Ca_2_Cu_3_O_10–_*_x_* (2223) tapes. The effects of sample characteristics, specimen mounting, measurement technique, and specimen damage were studied. The future development of a standard HTS measurement method is also discussed. Most of the evolution of this emerging technology has occurred in improvement of the performance of the conductors. The successful completion of this interlaboratory comparison is an important milestone in the evolution of HTS technology and marks a level of maturity that the technology has reached.

## 1. Introduction

### 1.1 Interlaboratory Comparison

An interlaboratory comparison is an essential part of the research needed to create a critical-current (*I*_c_) measurement standard for high temperature superconductors (HTS). A comparison can be used to evaluate the accuracy of existing laboratory practice and, if so designed, provide a technical basis for a future standard. Many of the technical considerations for this study are also relevant to large-scale application of HTS. The existing laboratory practice is based on previous low temperature superconductor (LTS) research and the additional known factors concerning HTS. This study was part of an international cooperative effort through the Versailles Project on Advanced Materials and Standards (VAMAS). Similar comparisons are being conducted independently in Japan [1] and Europe. The background research for the design of this study can be found in Ref. [2].

Two quantities that are common variables in critical-current measurements on superconductors are temperature and magnetic field. In this paper, the term temperature refers to the thermodynamic temperature, *T*, of the sample in units of kelvins, K. Also, the term magnetic field refers to the external applied magnetic field strength, *H*. For convenience and consistency with the practice of the superconductor industry, we express our magnetic field in terms of *μ*_0_*H* in units of tesla, T, where *μ*_0_ = 4π×10^−7^ H/m, the permeability of free space.

The history of critical-current measurements on superconductors has shown repeatedly that there is no substitute for an interlaboratory comparison to evaluate a measurement method and improve measurement consistency between different laboratories [3]. The subject of this paper is *I*_c_ measurements on HTS, and this is one of the first successful comparisons on HTS; however, there have been a number of comparisons on LTS. In many cases, when a group of laboratories conduct their first comparison, the results have had unacceptably large variations, and a second comparison was conducted with additional controls that lead to acceptable variations. For most applications of superconductivity, an acceptable variation in *I*_c_ measurements is approximately a coefficient of variation (the standard deviation divided by the average value) of 2 % to 4 %. This is not parts-per-million and thus it sounds easy enough; however, there are numerous effects that can each change the measured result by several percent [4]. An American Society for Testing and Materials (ASTM) standard for *I*_c_ measurements in LTS was created in 1982 [5] and has served as a guideline for many years. There is also a Japanese Industrial Standard (JIS) for *I*_c_ measurements in LTS. In 1984, a National Bureau of Standards (NBS) Standard Reference Material (SRM-1457) for the measurement of *I*_c_ [6] was created using a Nb-Ti (LTS) wire. This SRM allows a laboratory to test its *I*_c_ measurement system; however, the SRM does not test additional sources of variability among other superconducting materials, such as Nb_3_Sn (LTS) and HTS. The International Electrotechnical Commission (IEC) Council established a Technical Committee, TC90, in 1989 to create international standards for superconductivity. The VAMAS effort provides the technical basis for future IEC standards.

Two fairly recent *I*_c_ comparisons on LTS samples are summarized here to put this HTS study in perspective. A VAMAS comparison of *I*_c_ measurements on Nb_3_Sn conductors was started in 1986 and a complete summary of results from two comparisons was published in 1995 in a special issue of *Cryogenics* [7]. The results of the first VAMAS LTS comparison had unacceptably large variations; for example, on the best wire, the coefficient of variation was 8 %. Nb_3_Sn results from this comparison and the following comparisons were reported at 12 T and 4.2 K. In the second VAMAS LTS comparison, a detailed procedure was followed by all participating laboratories, and the variation for the same wire as above was reduced to 2.2 %. A similar learning curve was repeated in an International Thermonuclear Experimental Reactor (ITER) comparison of *I*_c_ measurements on Nb_3_Sn conductors which started in 1994. The ITER comparison involved a different set of international laboratories than the VAMAS comparison, although some laboratories participated in both comparisons. Some of the laboratories in the first ITER comparison used a common method which resulted in an acceptable coefficient of variation of measured *I*_c_ was 3 % to 4 % (the range of measured *I*_c_ was 18 %) [8]. The variations among the laboratories that did not use a common method was an unacceptable coefficient of variation of 10 % to 14 % (36 % *I*_c_ range). In the second ITER comparison, all of the participants used this common method, which resulted in acceptable variations, with the largest *I*_c_ range on a given sample being 3.4 % [9].

The critical current of a superconductor is one of its most important performance parameters, but *I*_c_ is difficult to measure accurately, and these measurements are often subject to scrutiny and debate. This is especially true for measurements on HTS specimens, where many factors can cause variability. For the purpose of this discussion, we have separated the sources of variability in critical-current measurements into four groups: sample, mounting, measurement, and damage. Sample variability includes sample inhomogeneity, *I*_c_ repeatability, and hysteresis of magnetic field and temperature. Mounting variability includes solder temperature, bonding agent, and substrate material. Measurement variability includes the general procedure, as well as repeatability and accuracy of voltage, current, magnetic field, temperature, and tap separation measurements. Damage variability includes thermal cycling, time, handling, and shipping. These are only partial lists of possible sources of variability. Although all of these effects are documented here, results were not always definitive because of the many concurrent effects and the limitation of time.

Two samples of Ag-sheathed Bi_2_Sr_2_Ca_2_Cu_3_O_10−_*_x_* (2223) tapes from different U.S. manufacturers, which we call Sample X and Sample Y, were studied. Twenty-nine specimens, each 42 mm long, from each sample were tested. In an attempt to isolate different sources of variability, the specimens were divided into different classes, with each class designed to isolate one or two sources of variability. The 29 specimens from each sample were divided into 6 classes, A to F, depending on four different parameters: routing of the specimens through the laboratories, and whether the specimens were premeasured, premounted, or preinstrumented. Table 1 gives the definition of the six classes. We routed specimens two different ways in this comparison. Some specimens were *series-routed*, meaning that they were sent from NIST, measured at each of the five U.S. laboratories in sequence, and then returned to NIST. Other specimens were *parallel-routed*, meaning that they were sent from NIST, measured by a single participating laboratory, and then returned to NIST. In the parallel routing, each laboratory measured different specimens, and no individual specimen was sent to more than one laboratory other than NIST. *Premeasured* specimens were measured by NIST before being sent out to any of the laboratories. *Premounted* specimens were bonded at NIST with a glass filled epoxy to a substrate material. Two commonly used substrate materials, G-10 (fiberglass-reinforced epoxy) and brass, were used to determine whether the substrate material made a difference in the measurement. *Preinstrumented* specimens had the voltage (1.5 cm tap separation) and current leads soldered to the specimen before shipment from NIST. A subset of this class is those specimens that had pressure current contacts and soldered voltage taps, and were measured on G-10 but were not bonded to it. This procedure of mounting allowed NIST to premeasure the specimen but also allowed the laboratories to mount the specimens using their own techniques.

**Fig. 1:**
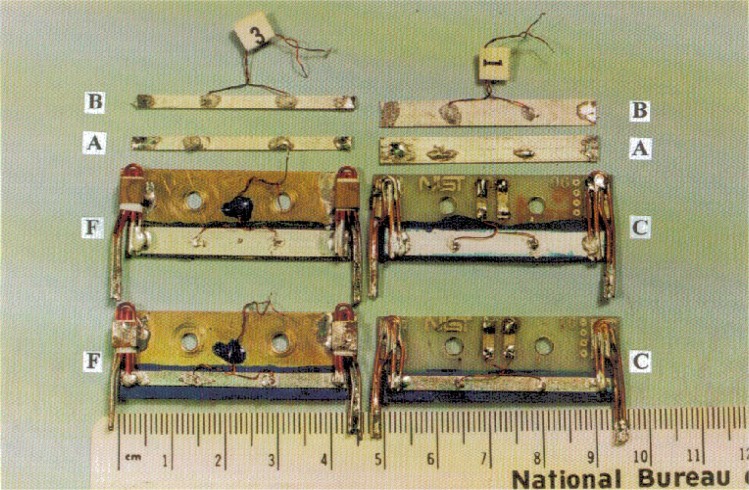
Photograph of specimens from Classes A, B, C and F and Samples X and Y, after they were returned from the laboratory that measured them.

**Fig. 2:**
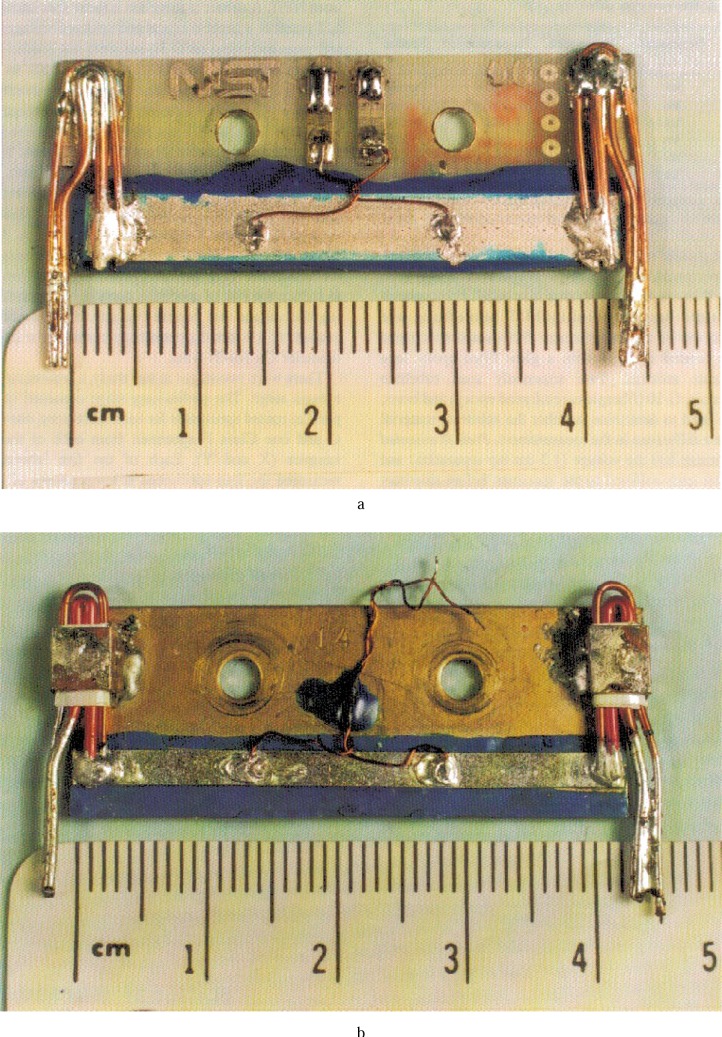
Photographs of two specimens after they returned from the laboratory (a) Sample X specimen from Class C and (b) Sample Y specimen from Class F.

Figures 1, 2a, and 2b are photographs of a few specimens after they returned from the laboratories. Figure 1 shows Class A, B, C and F specimens, one from each sample. Figure 2a shows a close up view of a Sample X specimen from Class C, and Fig. 2b shows a close up view of a Sample Y specimen from Class F. These figures illustrate how the specimens were mounted on the substrates. This method of mounting was designed to reduce the risk of specimen damage by strain-relieving the voltage and current contacts. The current lead consists of a 1 mm diameter copper wire and a 0.5 mm diameter Cu/Nb-Ti wire.

There were two stages in this study, a preliminary and a main stage. The preliminary stage consisted of four parallel routed specimens for each laboratory, one Class C and one Class F specimen from each of the two samples (X and Y). Each of the five laboratories measured the four specimens at temperatures of 4.2 K and 77 K with zero applied magnetic field. Zero applied magnetic field measurements may have been made in small remnant fields and were made in the self-field created by the specimen current. The preliminary stage gave us feedback, which let us conduct the main stage more efficiently. We determined that most specimens could tolerate shipment from one laboratory to another. We also discovered that some specimen bubbling would occur (this will be explained later), possibly due to rapid warming of the specimen, and therefore we asked the laboratories to report how they cooled and warmed the specimens that they measured in the main stage.

The main stage of the experiment had both series-routed (D and E) and parallel-routed (A, B, and C) specimens. One specimen from each of Class A, D, and E from each sample was measured in zero applied magnetic field at temperatures of 4.2 K and 77 K. The *I*_c_ measurements at these two temperatures were made with the specimens immersed in a liquid cryogen (liquid helium and liquid nitrogen). One specimen from each of Classes B, C, D, and E from each sample was measured as a function of magnetic field at temperatures of 4.2 K and 77 K. The field measurements were requested at fields of (0, 0.1, 0.2, 0.5, 1, 2, 4, and 8) T at 4.2 K and (0, 0.005, 0.01, 0.02, 0.05, 0.1, 0.2, 0.5, and 1) T at 77 K. Each specimen had a unique alphanumeric designation, and the laboratories were given specific measurement instructions for each specimen. Magnetic field measurements were made with the applied magnetic field perpendicular to current *I* and applied magnetic field parallel to the wide face of the tape. In order to avoid hysteretic effects, we instructed the laboratories to measure only with increasing magnetic field.[10]

In this comparison, each laboratory, excluding NIST, measured a total of 18 specimens (10 in zero applied magnetic field only). We chose a large number of specimens and the complex class and routing system in order to obtain sufficient data to be more likely to have statistically meaningful results. NIST measured a total of 48 specimens, and each specimen was measured two or three times.

### 1.2 NIST Measurements

The estimated expanded uncertainty (coverage factor *k* = 2, and thus a two-standard-deviation estimate) of the NIST critical-current measurements is ± 1 %. In order to remove variability due to sample inhomogeneity, NIST premeasured all specimens except those from Class A. This allowed normalization of all subsequent measurements to the premeasured values. Most of the *I*_c_ measurements reported here were made using an electric field strength criterion of 1 μV/cm.

**Fig. 3:**
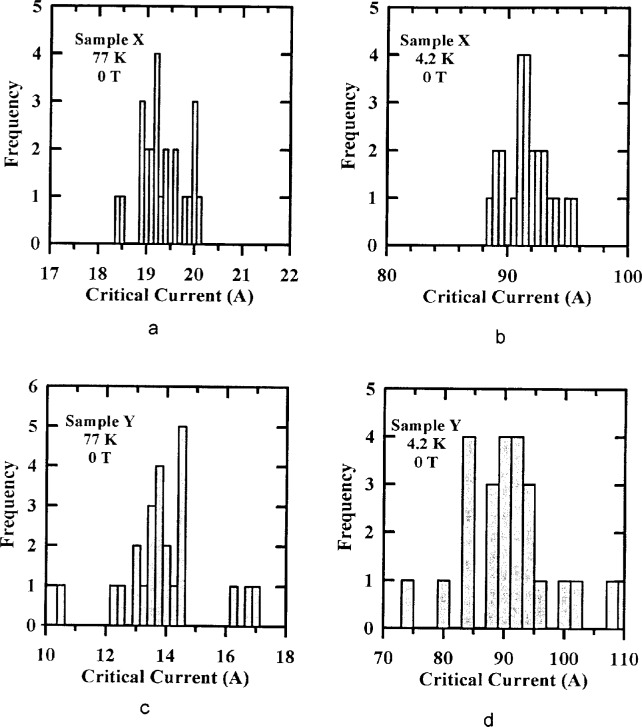
Distribution of NIST’s initial critical current measurements on 24 specimens at 1 μV/cm and 0 T. Specimens were from the preliminary and main stages of the experiment for (a) Sample X at 77 K, (b) Sample X at 4.2 K, (c) Sample Y at 77 K, (d) Sample Y at 4.2 K.

Figures 3a to 3d are histograms of NIST’s initial critical-current measurements from the preliminary and main stages of the experiment for specimens from Samples X and Y at temperatures of 77 K and 4.2 K with zero applied magnetic field. These 24 specimens were from classes B, C, D, E, and F. These classes include a wide variety of mounting parameters, including B specimens which had pressure current contacts to allow mounting of the specimens by the laboratories. There were no apparent trends between the specimen class and their locations within the distribution. This indicates that the difference in substrate material (G-10 and brass) was not significant. Table 2 gives summary statistics for the NIST data shown in Figs. 3a to 3d.

The Sample X histograms show remarkably low variability, with a coefficient of variation of 2.5 % at 77 K and 2.0 % at 4.2 K. The Sample Y specimens had acceptably low variability, with a coefficient of variation of 8.6 % at 77 K and 7.0 % at 4.2 K. One specimen from Sample Y was removed from the statistics because it was possibly an outlier. Since most of the specimens in the comparison were premeasured and the data were normalized based on these measurements, the effect of this variability was reduced. We are assuming that most of this variability is due to sample inhomogeneity rather than the NIST measurement and mounting variability.

The zero-field value of *I*_c_ for both samples was between 12 A and 20 A at 77 K and between 80 A and 110 A at 4.2 K, excluding the Sample Y outlier. The abruptness of the transition from the superconducting to the normal state is characterized by the *n*-value which is a fit parameter in an empirical equation for the voltage-current characteristic of the superconductor. The *n*-values for these two samples near the *I*_c_ criterion of 1 μV/cm were about 30 in zero field at both temperatures. These n-values are relatively high indicating fairly abrupt transitions and well-defined critical currents.

**Fig. 4:**
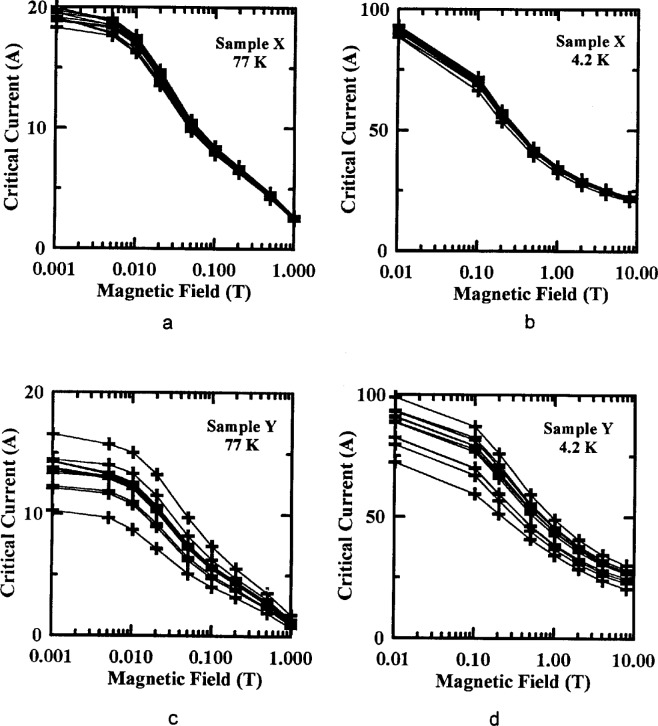
NIST’s initial critical current measurements versus magnetic field on 12 specimens at 1 μV/cm. Specimens were from the preliminary and main stages of the experiment for (a) Sample X at 77 K (b) Sample X at 4.2 K, (c) Sample Y at 77 K, (d) Sample Y at 4.2 K.

Measurements of the effect of applied magnetic field on *I*_c_ were also studied. Typical *I*_c_ versus magnetic field curves from NIST data are shown in Figs. 4a to 4d for Samples X and Y at 77 K and 4.2 K. A semi-logarithmic scale was used. To avoid scaling problems, 0.001 T was designated zero applied field at 77 K, and 0.01 T was designated zero applied field at 4.2 K. Table 3 gives coefficients of variation for the NIST *I*_c_ measurements versus magnetic field. The coefficient of variation increased only slightly with increasing magnetic field except for Sample Y at 77 K, because of the increased field sensitivity of this sample. The magnetic field dependencies of *I*_c_ for these specimens formed a family of well behaved curves which allowed us to normalize subsequent data to the initial measured values.

### 1.3 Severe Specimen Damage

After the data were compiled we identified three sources of variability caused by severe damage.

1. Bubbles in the Ag sheath between or near the voltage taps. These bubbles were probably due to rapid evaporation of liquid cryogen, which had seeped into the specimen during the measurements, when the specimen was extracted from the cryogen.
2. Specimens coming loose from the substrate. This occurred only on the brass substrates and could be due to poor bonding or differential thermal contraction. We had prepared the brass substrate for bonding the specimen to it by roughing the substrate surface with sandpaper and degreasing it.
3. Mechanical specimen damage, possibly caused by bumping the specimen against the measurement Dewar during insertion or extraction.

Most of the specimens (10 of 13) with a measured critical-current degradation greater than 10 % had visible severe damage. We could distinguish these severe sources of damage variability from the other sources of variability by correlating visible specimen anomalies with reductions in critical current. These sources of specimen damage could be reduced by preliminary thermal cycling to identify and subsequently eliminate specimens that are prone to bubble, eliminating brass as a substrate material, and protecting the specimens during measurements so they cannot inadvertently bump against the Dewar. Data taken on damaged specimens are treated as outliers in the analysis presented below and they are omitted from the statistics.

## 2. Primary Results—Parallel Routing

### 2.1 Zero Applied Magnetic Field

Many of the plots throughout this paper are followed by another plot containing a subset of the same data, but with the *y*-axis expanded to focus on data from specimens that were not damaged.

**Fig. 5:**
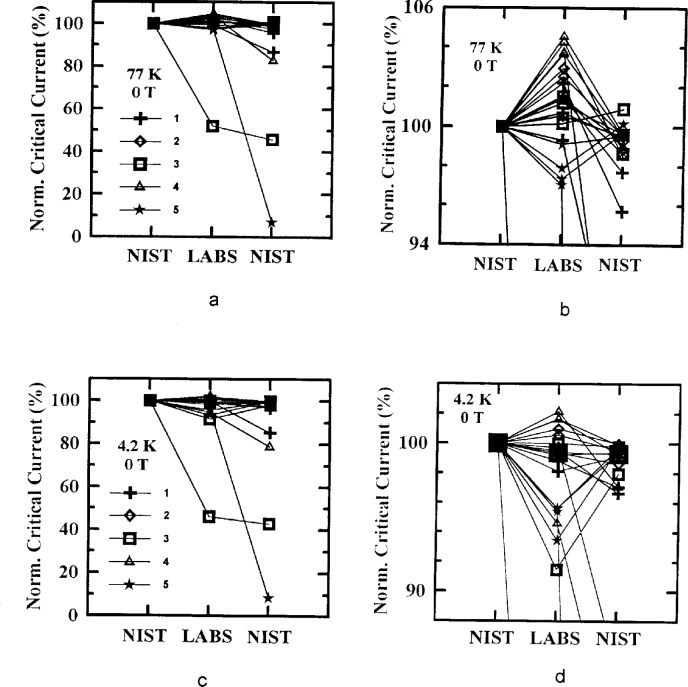
Normalized critical current versus measurement laboratory at 1 μV/cm and 0 T for 20 parallel specimens from Samples X and Y in the preliminary stage. The symbols represent the different laboratories. Specimens measured at (a) 77 K, (b) 77 K with expanded scale, (c) 4.2 K, (d) 4.2 K with expanded scale.

**Fig. 6:**
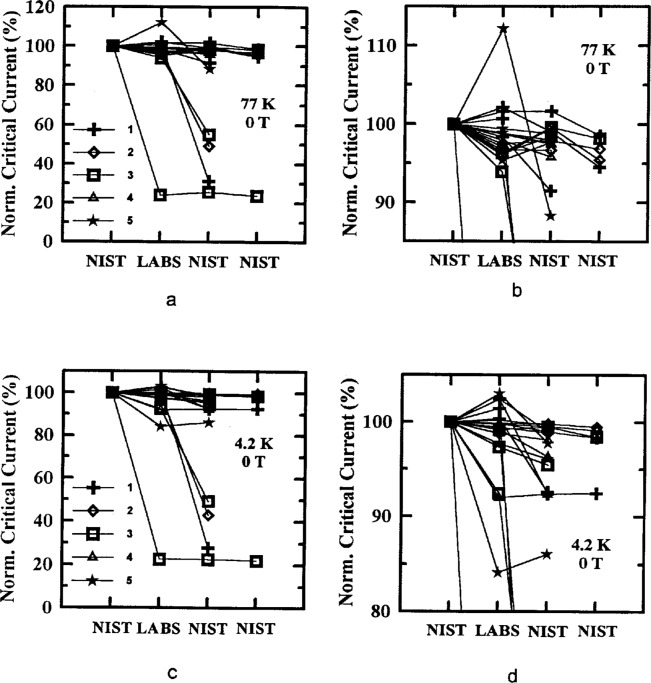
Normalized critical current versus measurement laboratory at 1 μV/cm and 0 T for 20 parallel specimens from Samples X and Y in the main stage. Specimens measured at (a) 77 K, (b) 77 K with expanded scale, (c) 4.2 K, (d) 4.2 K with expanded scale.

Figures 5a to 5d and 6a to 6d show the normalized critical current at 1 μV/cm and zero applied magnetic field at temperatures of 77 K and 4.2 K. Figures 5a to 5d contain data from the preliminary stage (Class C and F) while Figs. 6a to 6d contain data from the main stage (Class B and C). Different specimens were used in the two stages. All of the plots include data from both Sample X and Y. The *x*-axis shows the sequence in which measurements were made. The specimens were first measured at NIST, then at the five laboratories in parallel, and then remeasured at NIST. Some specimens were remeasured twice at NIST, in which case NIST appears twice near the right end of the *x*-axis. The normalization was done with respect to NIST’s initial measurements. The laboratories are denoted by numbers 1 to 5, and consistent data symbols are used throughout this paper. Lines on the plots connect data points for specific specimens.

The plots indicate that most specimens did not degrade by more than 5 %, while four or five specimens significantly degraded by the time they were returned to NIST for remeasurement. These data indicate that only one specimen was significantly degraded between NIST’s initial measurement and the laboratory measurement in both stages of the experiment, by coincidence. Most of the specimens that degraded to less than 95 % of the original NIST measurement had visible damage. Tables 4 and 5 give the summary statistics for specimens from the preliminary and main stages of the comparison. Some of the specimens were considered outliers and were omitted from the statistics.

The plots also indicate that the variability of the measurements of the participating laboratories was low, about a 10 % spread in the data, excluding outliers. There was a slight increase in the variability of the measurements in the main stage, which can be seen by comparing Tables 4 and 5. This could be due to the addition of Class B specimens in the main stage, which had to be mounted by the laboratories. The variability of the laboratory measurements is larger than the variability of the final NIST measurements due to the differences among the measurement systems of the laboratories. This part of the experiment was designed to focus on the measurement bias and variability of individual laboratories. For example, Laboratory 4’s measurements had a positive bias, while Laboratory 5’s measurements had a negative bias. A laboratory bias is computed relative to the initial NIST measurements. This does not imply that NIST measurements are without bias, they were merely used as a convenient baseline. It is a significant accomplishment that most of these 40 specimens degraded by less than 3 %, on average, between the initial and final NIST measurements, and that the laboratory average was within 2 % of the initial NIST measurement. However, it may be possible to reduce these biases once they are identified.

### 2.2 Critical Current Versus Magnetic Field

**Fig. 7:**
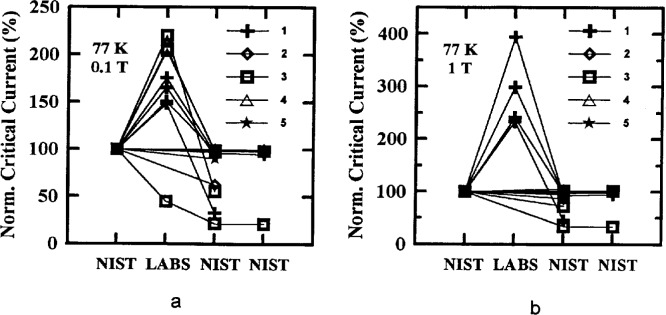
Normalized critical current versus measurement laboratory at 77 K and 1 μV/cm for 20 parallel specimens from Samples X and Y in the main stage at a magnetic field of (a) 0.1 T and (b) 1 T.

**Fig. 8:**
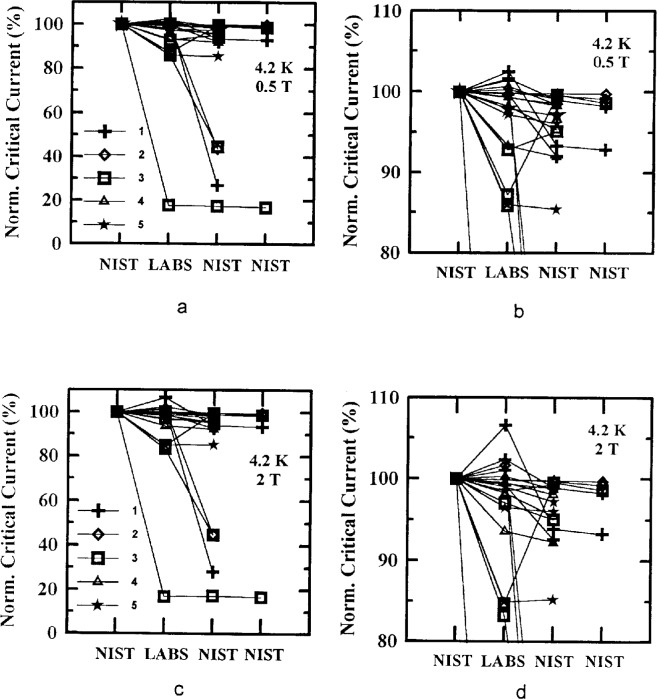
Normalized critical current versus measurement laboratory at 4.2 K and 1 μV/cm for 20 parallel specimens from Samples X and Y in the main stage at a magnetic field of (a) 0.5 T, (b) 0.5 T with expanded scale, (c) 2 T, (d) 2 T with expanded scale.

**Fig. 9:**
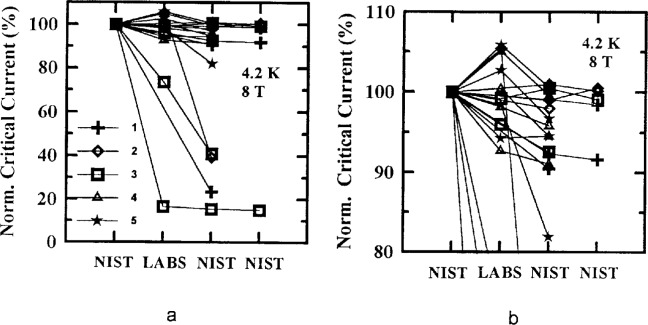
As in Fig. 8 except at a magnetic field of (a) 8 T and (b) 8 T with expanded scale.

Critical-current measurements versus field were made on four of the parallel specimens in the main stage, two from each of Samples X and Y at both 77 K and 4.2 K. Measurements at 77 K were requested at fields of (0, 0.005, 0.01, 0.02, 0.05, 0.1, 0.2, 0.5, and 1) T. Measurements at 4.2 K were requested at (0, 0.1, 0.2, 0.5, 1, 2, 4 and 8) T. Figures 7a and 7b show normalized critical-current measurements at 77 K, 1 μV/cm, and several magnetic fields, and Figs. 8a to 8d, 9a, and 9b show normalized critical-current measurements at 4.2 K, 1 μV/cm, and several magnetic fields. Tables 6 and 7 give summary statistics for these measurements at 77 K and 4.2 K. Some of the data points were treated as outliers and were not used in calculating the statistics.

Three of the five laboratories did not make *I*_c_ measurements in a magnetic field at 77 K, for various reasons. NIST measurements were still included on all 20 specimens. Results from the two laboratories that made these measurements were significantly different from the NIST measurements. The coefficients of variation for the two laboratories were ranging from 28.7 % to 74.1 %, much higher than the zero-magnetic-field coefficient of variation, which was 4.4 %. At 0.1 T, Laboratory 1 measured more than 50 % and Laboratory 3 measured more than 100 % higher than the NIST baseline. At 1 T, Laboratory 1 was the only laboratory to make measurements and they were 100 % to 300 % high. These high measurements were likely due to calibration errors that will be discussed later in this paper. The same four specimens that indicated damage at zero applied field showed signs of damage in these measurements. One of the damaged specimens was not measured at field by any laboratory other than NIST.

The variability in the field measurements at 4.2 K was quite low, excluding damaged specimens which were considered outliers. The same four specimens that indicated damage at zero field showed damage with nonzero applied magnetic field. Most of the specimens were degraded by less than 10 % when they were returned to NIST. The coefficient of variation for the laboratories was larger with an applied magnetic field (4.5 % to 6.8 %) than without (3.2 %). The coefficient of variation at 2 T was the largest; however only four of the five laboratories measured critical current at 8 T which may have affected the coefficient of variation at 8 T. The bias of each laboratory is fairly evident in the plots with the expanded scales. Laboratory 3 had significant negative bias for most of these measurements. Any magnetic field calibration errors in the 4.2 K data were much less than observed in the 77 K data.

## 3. Primary Results—Series Routing

Eight of the specimens in this interlaboratory comparison were series-routed, that is, they were sent from NIST and were measured at each of the five laboratories in sequence (indicated by laboratory number) and then returned to NIST for remeasurement. All eight specimens were measured at zero applied magnetic field, but only four of the specimens were measured versus magnetic field. This routing pattern allowed for all the participating laboratories to measure the same specimen, but it also introduced more damaged specimens and took longer to complete.

**Fig. 10:**
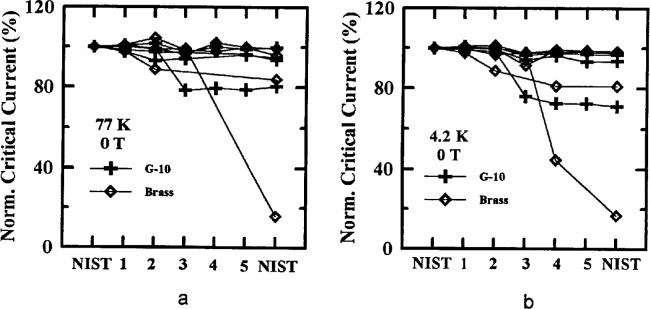
Normalized critical current versus measurement laboratory at 1 μV/cm and 0 T for 8 series specimens from Samples X and Y at temperatures of (a) 77 K and (b) 4.2 K.

**Fig. 11:**
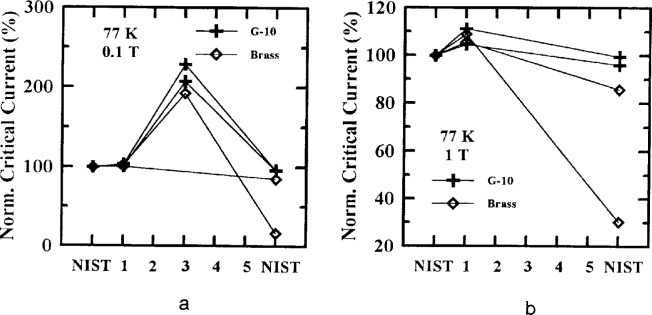
Normalized critical current versus measurement laboratory at 77 K and 1 μV/cm for 4 series specimens from Samples X and Y at magnetic fields of (a) 0.1 T and (b) 1.0 T.

**Fig. 12:**
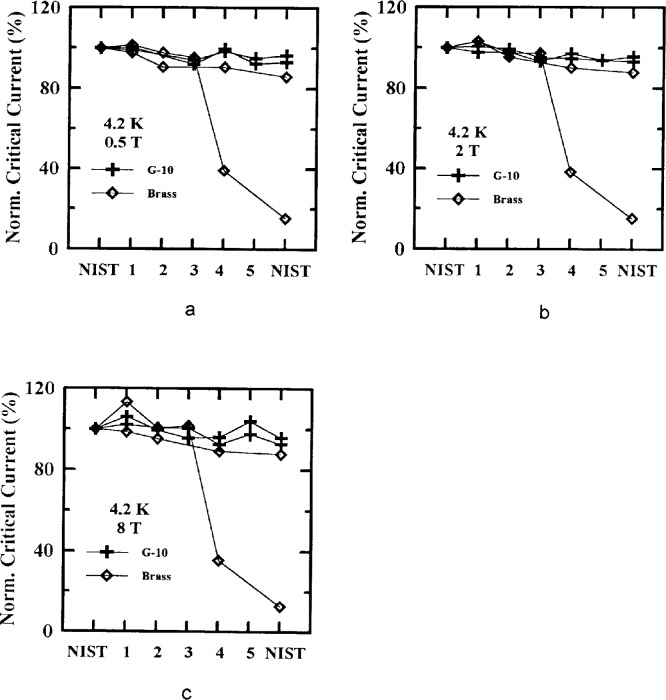
Normalized critical current versus measurement laboratory at 4.2 K and 1 μV/cm for 4 series specimens from Samples X and Y at magnetic fields of (a) 0.5 T, (b) 2.0 T, (c) 8.0 T.

Four specimens were from Sample X and four from Sample Y, with four mounted on brass and four mounted on G-10. Figures 10a, 10b, 11a, 11b, 12a, 12b, and 12c show normalized critical current for series-routed specimens at 1 μV/cm. Figures 10a and 10b were at zero applied magnetic field and temperatures of 77 K and 4.2 K. Figures 11a and 11b were at 77 K and applied magnetic fields of 0.5 T, 2 T, and 8 T. The *x*-axis shows the laboratory sequence (1 to 5) with every specimen starting and ending at NIST. Some of the laboratories chose not to make measurements on some of the specimens (possibly because of observable damage), and therefore some of the lines do not have data points at every laboratory.

Five out of the eight specimens measured in zero applied magnetic field degraded by less than 7 %, while three specimens (two on brass and one on G-10) degraded significantly by the time they were returned to NIST. The five specimens without significant damage showed a trend of slight degradation with time during the series routing.

As in the parallel-routed specimens, only two of the laboratories, other than NIST, made field measurements at 77 K. The measurements of one of these laboratories were more than 100 % higher than NIST’s initial measurements. This was due to a magnetic field calibration error and will be discussed below. The other laboratory measured up to 12 % high and will also be discussed below. Only one of the specimens had significantly degraded by the time it was returned to NIST. This specimen indicated significant degradation at both temperatures and all magnetic fields.

The field measurements at 4.2 K showed a similar trend to the zero applied magnetic field measurements.

## 4. Supplementary Results

### 4.1 Laboratory Mounting Variability

Variability caused by different mounting and measurement techniques is best demonstrated with measurements on specimens from Classes A and B. Since Class A specimens had no preinstrumentation or premeasurement, they were susceptible to wider variability. Class B specimens allowed the laboratories to mount the specimens using their own techniques but still allowed NIST to premeasure the specimen and therefore normalize the measurements of the laboratories. This method allowed us to get a better view of each laboratory’s bias and variability. Table 8 gives the summary statistics for the Class A and Class B specimens.

**Fig. 13:**
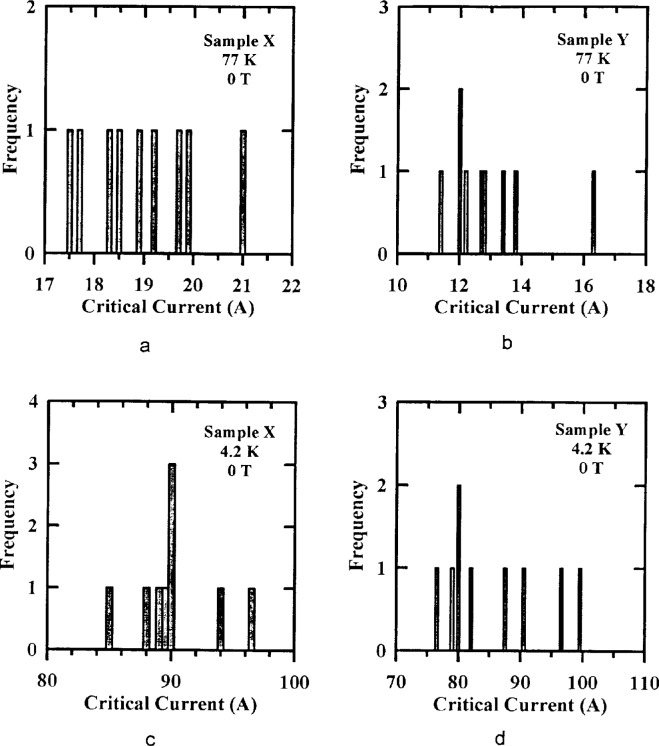
Distribution of measurements of the five laboratories on Class A and B specimens at 1 μV/cm and 0 T for (a) Sample X at 77 K, (b) Sample Y at 77 K, (c) Sample X at 4.2 K, (d) Sample Y at 4.2 K.

Figures 13a to 13d show the distribution of the measurements of the laboratories with zero applied magnetic field on Class A and B specimens from Samples X and Y and at 77 K and 4.2 K. There was no apparent trend in the separate distributions of Class A and B specimens. These distributions are slightly wider than the distributions of NIST’s initial data shown in Table 2 due to the additional source of variability introduced by the laboratory mounting, although the NIST measurement data included data from specimens mounted in various ways. The coefficients of variation for the NIST distributions ranged from 2 % to 9 % while the coefficients of variation for the laboratories distributions ranged from 4 % to 11 %. The observed trend was that the coefficients of variation for the laboratories distributions were higher by about 2 % in each case.

In Figs. 13a to 13c, a Class B specimen is missing because one of the laboratories did not measure it. Also, a Class A specimen was omitted from the Sample Y, 4.2 K statistics because it was considered an outlier.

### 4.2 Specimen Variability as a Function of Time

One of the extra variables studied in this comparison was the degradation in *I*_c_ as a function of time. This degradation could be due to specimen handling, mounting and unmounting, and time. The comparison spanned almost a year between NIST’s first measurement to NIST’s last remeasurement. Extra measurements were made on some of the Class C specimens to obtain more data over a longer period of time.

**Fig. 14:**
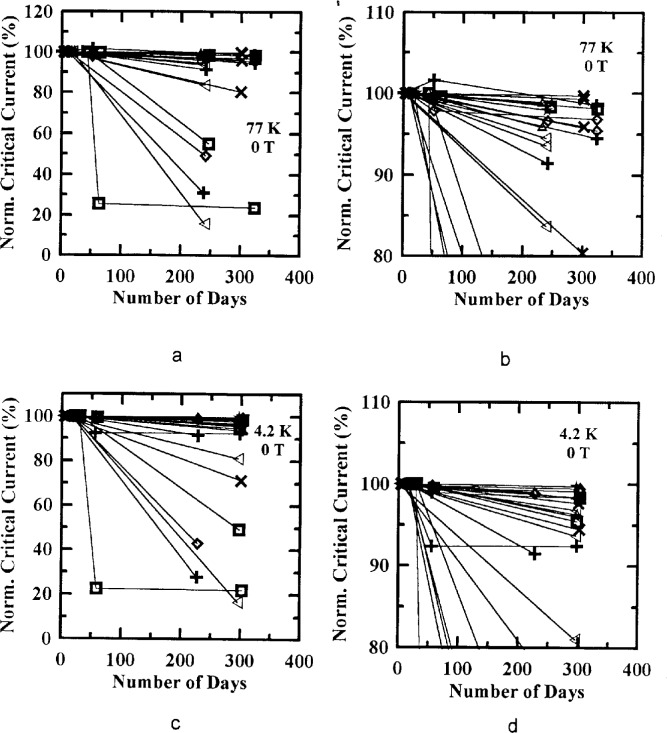
NIST’s normalized critical current as a function of elapsed time from the first NIST measurement at 1 μV/cm and 0 T at temperatures of (a) 77 K, (b) 77 K with expanded scale, (c) 4.2 K, (d) 4.2 K with expanded scale.

Figures 14a to 14d shows NIST’s normalized *I*_c_ measurements at 77 K and 4.2 K on 28 parallel and series (B, C, D, and E) specimens at zero applied magnetic field, as a function of the elapsed time from the first NIST measurement. In most cases, the degradation over time was not severe. The curves connecting the various measurements on a specific specimen (those that had not been severely damaged) had zero or small slope. Most of the final measurements are within 10 % of NIST’s first measurement even after a year.

### 4.3 Electric Field Strength Criterion

The laboratories were asked to measure the critical current at three electric field strength criteria, 0.1μV/cm, 1 μV/cm, and 10 μV/cm. Throughout this paper we have given data at only 1 μV/cm. Many of the laboratories did not make measurements at 0.1 μV/cm, possibly because of a low signal-to-noise ratio. Most of the laboratories made measurements at 1 μV/cm and 10 μV/cm. Table 9 gives summary statistics for the critical current at 10 μV/cm at both 4.2 K and 77 K for the 20 specimens in the preliminary stage. The coefficient of variation was slightly lower for the laboratories and NIST at both temperatures compared to the 1 μV/cm results given in Table 4. For example, the coefficient of variation for the laboratories at 4.2 K was 3.03 % at 1 μV/cm and 2.95 % at 10 μV/cm. This could be due to the larger signal at 10 μV/cm.

**Fig. 15:**
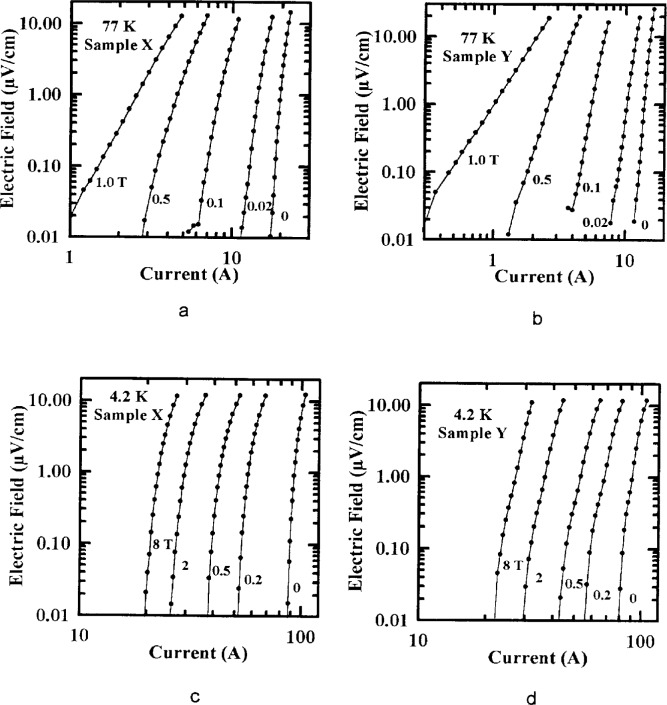
NIST’s electric field data versus current at various magnetic fields for one specimen from (a) Sample X at 77 K, (b) Sample Y at 77 K, (c) Sample X at 4.2 K, (d) Sample Y at 4.2 K.

Typical NIST electric field strength (*E*) versus *I* curves at various magnetic fields are given in Figs. 15a to 15d to illustrate the shapes of the curves. The typical noise was about 0.01 μV/cm. The transition was fairly abrupt and the shape provided well-defined values of *I*_c_ at all measured fields and temperatures.

### 4.4 Critical-Current Repeatability

A phenomenon that was observed in this interlaboratory comparison was the tendency for critical-current values to increase or decrease with repeated determinations. If *I*_c_ only increased with each determination, it would be called training. The critical current also seemed to return close to its initial value after a thermal cycle. This suggests that the critical current in these specimens has a hysteresis with multiple determinations, similar to the magnetic field hysteresis. We measured four specimens, two from each sample, at both 4.2 K and 77 K. Seven determinations of *I*_c_ were made at constant field and temperature to observe this effect.

**Fig. 16:**
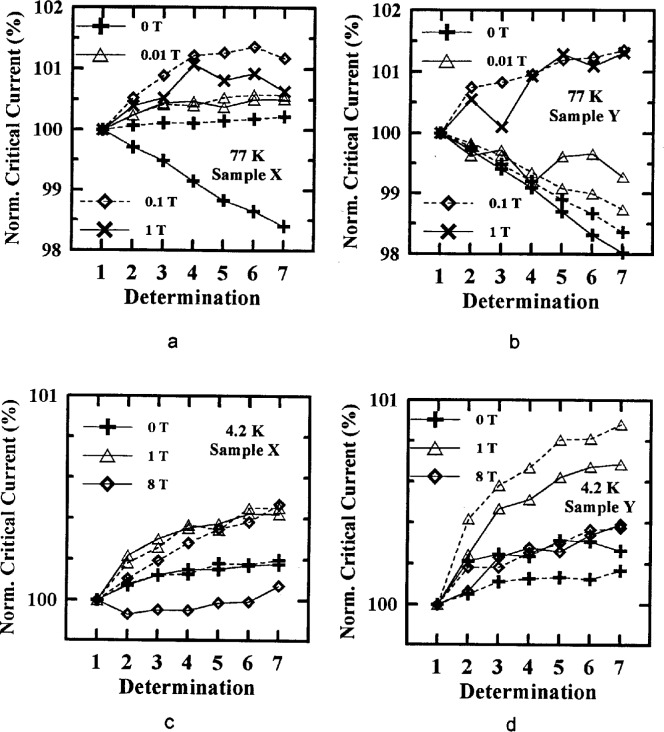
Multiple determinations of NIST’s normalized critical current at various magnetic fields for two specimens from (a) Sample X at 77 K, (b) Sample Y at 77 K, (c) Sample X at 4.2 K, (d) Sample Y at 4.2 K.

Figures 16a to 16d show multiple determinations on two specimens from Samples X and Y on G-10. Figures 16a and 16b show specimens measured at 77 K and three magnetic fields. One specimen had multiple determinations at magnetic fields of 0 T, 0.01 T, and 1 T while the other specimen had multiple determinations at magnetic fields of 0 T, 0.01 T, and 0.1 T. Figures 16c and 16d show specimens measured at 4.2 K and three magnetic fields, 0 T, 1 T, and 8 T. The different line types distinguish the two specimens and the different symbols distinguish different magnetic fields.

Multiple determinations made at 77 K had specimens with increasing and decreasing critical current, while all of the multiple determinations made at 4.2 K had specimens with increasing critical current. At 77 K, the *I*_c_ at higher magnetic fields tended to increase more, while at 4.2 K, the middle field, 1 T, had the greatest increase.

Repeatability depends on both magnetic field and temperature. The same specimen that had an increase or decrease in *I*_c_ with determinations at 4.2 K may or may not have an increase or decrease in *I*_c_ with determinations at 77 K. The increase or decrease in *I*_c_ for some of the specimens flattened out with higher determinations, and some showed no sign of flattening out. The increases and decreases ranged between 1 % and 2 %, which could be a contributing factor to variability in the measurements. The NIST data that were used in the rest of the comparison included an average of three determinations. This was sufficient for this study, but if more accurate results are needed, further repeatability studies will be needed.

### 4.5 Measurement Errors

All of the significant *I*_c_ errors in this interlaboratory comparison were found in *I*_c_ data versus magnetic field. Critical-current measurements versus field at 77 K seemed to be particularly difficult for the laboratories. Three of the laboratories did not make any 77 K measurements versus magnetic field. Two of the laboratories had significant *I*_c_ errors in at least half their data. In both cases, these *I*_c_ errors were thought to result from errors in magnetic field calibration. This was indicated by the fact that the *I*_c_ data at zero field were close to NIST results and the error in *I*_c_ increased with increasing field.

**Fig. 17:**
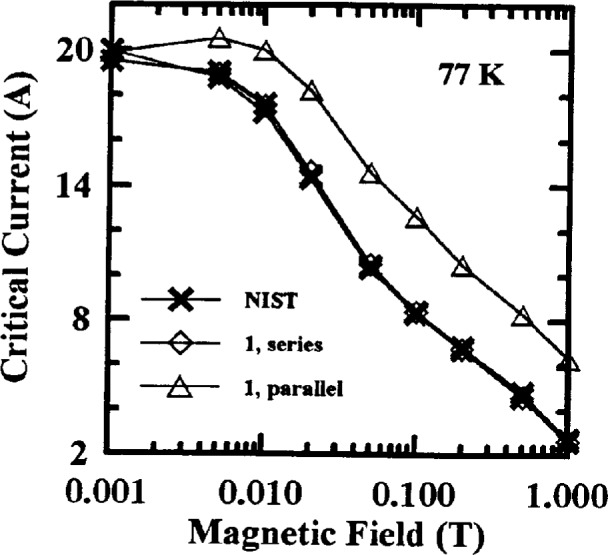
Critical current measurements versus magnetic field made by Laboratory 1 at 77 K and 1 μV/cm for a series and a parallel specimen. NIST data on these two specimens are also included for comparison.

Laboratory 1’s *I*_c_ data were high at 77 K on their parallel routed specimens with respect to NIST data, and somewhat high on their series-routed specimens. Figure 17 shows Laboratory 1’s *I*_c_ measurements versus magnetic field on a series-routed and a parallel-routed specimen from Sample X. NIST data on these two specimens are included for comparison. The average bias of *I*_c_ measurements at 1 T for Laboratory 1 was 192 % high for parallel specimens and 8 % high for series specimens. This laboratory may have used different measurement systems for these two sets of specimens, which were measured a few weeks apart.

**Fig. 18:**
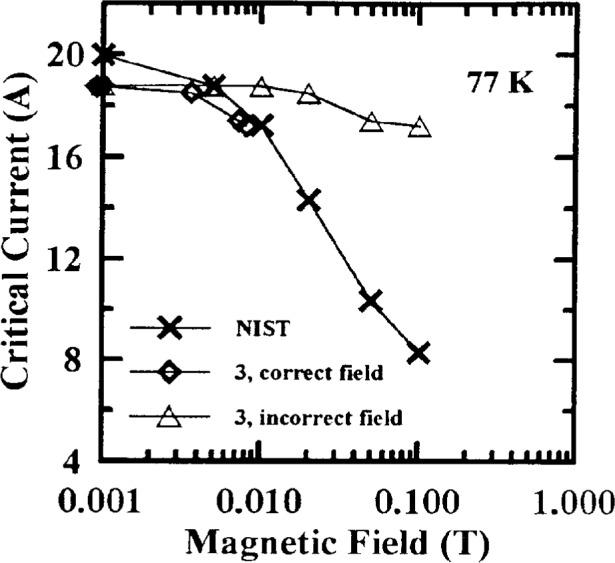
Critical current measurements versus magnetic field on one specimen made by Laboratory 3 at 77 K and 1 μV/cm, with both the corrected and uncorrected magnetic field. NIST data on the same specimen are also included for comparison.

This comparison also revealed that Laboratory 3 was off by a factor of 12 in magnetic field calibration at 77 K and therefore its *I*_c_ measurements were off by up to 100 %. Figure 18 shows the original *I*_c_ measurements made by Laboratory 3 with the field calibration error. It also gives its original *I*_c_ measurements with the field calibration error corrected. NIST data are also included in this plot for comparison.

**Fig. 19:**
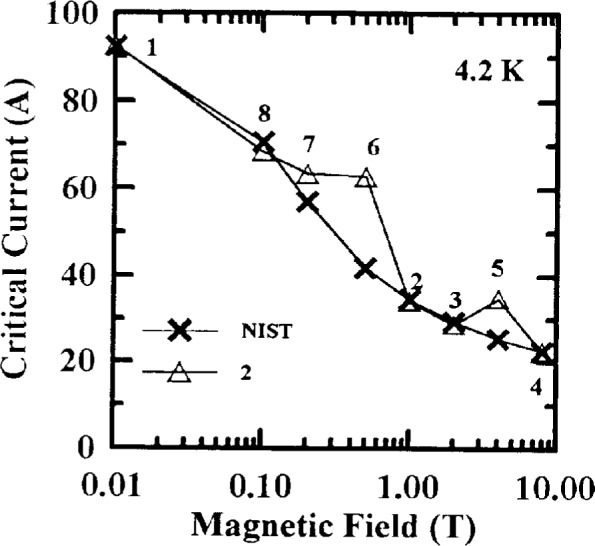
Critical current measurements versus magnetic field demonstrating hysteresis made by Laboratory 2 at 4.2 K and 1 μV/cm. The number near each point indicates the measurement sequence for Laboratory 2. NIST data on the same specimen are also included for comparison.

Critical-current measurements versus field at 4.2 K seemed to be less difficult. However, Laboratory 2 measured three of their specimens versus field at 4.2 K in a somewhat random order of magnetic fields. Figure 19 shows one set of its *I*_c_ measurements versus magnetic field. NIST data on these specimens are also included for comparison. The numbers above each data point indicate the order in which the data were taken. This is an example of the field hysteresis that occurs if a value of *I*_c_ is measured at a lower field after the sample was in a higher field. This demonstrates the importance of measuring *I*_c_ with monotonically increasing magnetic field.

## 5. Creating a Standard Technique for HTS Specimens

One of the goals of this interlaboratory comparison was to investigate what is needed to develop a standard *I*_c_ measurement practice for HTS specimens. The results of this comparison indicate that a more restrictive technique than that used by the participating laboratories may not be necessary, and a consensus standard could be developed. It should be emphasized that the specimens used in this interlaboratory comparison were fully sheathed Ag-Bi tapes; other HTS specimens may require more restrictions. Also, NIST had considerable control over the measurements made in this study, and the participating laboratories may have been more careful than usual in their measurements of these specimens. Table 10 gives the measurement details of each laboratory including cooling time from room temperature to 77 K, whether they precooled in liquid nitrogen before measuring in liquid helium, the cooling time between liquid nitrogen temperature and liquid helium temperature, whether they warmed the specimen above 120 K between 77 K and 4.2 K, how many critical-current determinations they made at each temperature and field, whether the specimen was covered to protect it from damage, and whether the specimen was exposed to air below the dew point temperature. It is interesting that all of the laboratories, except NIST, exposed the specimen below the dew-point temperature. This implies that it may not be necessary to isolate the specimen from the atmosphere. Most of the laboratories protected the specimen from damage with some sort of cover. Cooling times range from less than 1 min up to 25 min. Even with all of these variations, fairly consistent results were still obtained. Even though some specimens were damaged and some obvious measurement errors were made, there were no measurement techniques that led to damaged specimens or significantly biased results, relative to the NIST baseline, on every specimen.

Table 11 gives the laboratory mounting details for Class A and B specimens, including the composition of solder used, the melting point of the solder, the soldering iron temperature, and a brief description of how they mounted these specimens. A wide variety of soldering techniques were used by the various laboratories. There was also quite a bit of variability in mounting technique; however, consistent results were still obtained. The variability in the Class A and B specimens was still quite low, with coefficients of variation ranging from 3.7 % to 11.2 % depending on sample and temperature.

## 6. Summary

NIST acted as the central laboratory in this comparison by premounting and premeasuring most of the specimens, as well as remeasuring the specimens after they had been measured by the participating laboratories. The initial NIST measurements were made on specimens mounted in different ways (solder contacts or pressure contacts) using different substrates (G-10, brass, or none). Initial critical-current homogeneity for each sample was more than adequate for this comparison. Zero-magnetic-field coefficients of variation were 2.5 % at 77 K and 2.0 % at 4.2 K for Sample X and 8.6 % at 77 K and 7.0 % at 4.2 K for Sample Y. Since most of the specimens were premeasured, we were able to compare measurements without being limited by sample homogeneity. However, the observed sample homogeneity allowed more-definitive conclusions to be made.

Measurements in the preliminary stage of this comparison resulted in participating laboratory coefficients of variation of 2.3 % at 77 K and 3.0 % at 4.2 K. All 20 specimens from Sample X and Y were premounted and measured at zero applied magnetic field. Measurements in the main stage resulted in participating laboratory coefficients of variation of 4.4 % at 77 K and 3.2 % at 4.2 K. All 20 specimens from Sample X and Y were measured in zero applied magnetic field. Half of the specimens were premounted and half were laboratory mounted. This slight coefficient of variation increase in the main stage could be due to the additional variability introduced by the laboratory mounting.

Measurements at 4.2 K as a function of magnetic fields up to 8 T showed an increase in the coefficient of variation, with the largest coefficient of variation 6.8 %. Measurements at 77 K as a function of magnetic fields up to 1 T revealed serious magnetic field calibration errors. A number of laboratories did not make any field measurements at this temperature for various reasons. Laboratory coefficients of variation at 77 K were about 29 % at 0.1 T and 74 % at 1 T. The NIST remeasurements had coefficients of variation of about 3 % at these fields. These magnetic field calibration errors must be corrected at 77 K.

Series routing of specimens through the participating laboratories can be done; however, it should be attempted only if the comparison is not on a strict schedule. Parallel routing is more work for the central laboratory, but it is easier for the participating laboratories because there is less time constraint. This approach also takes less time to complete. The difference between G-10 and brass as a substrate material was not apparent in the *I*_c_ measurements; however, specimens on brass seemed more likely to separate from the substrate. In retrospect, we could have eliminated brass as a mounting material and had fewer specimens. Other parameters that were studied in this comparison were dependence on electric field strength criterion, *I*_c_ repeatability, mounting and measurement details, sequence of measurements versus magnetic field, and *I*_c_ degradation as a function of time. We observed that magnetic field and repeat determination hysteresis could be reversed by thermally cycling the specimen. The critical current usually returned within 0.2 % of its initial value after a thermal cycle.

## 7. Conclusions

- This interlaboratory comparison achieved acceptably low variations in *I*_c_ measurements on Ag-sheathed Bi_2_Sr_2_Ca_2_Cu_3_O_10−_*_x_* tapes as a function of magnetic field at 4.2 K and 77 K.
- Most of the specimens in this comparison tolerated multiple measurements.
- The significant sources of variability are: magnetic field calibrations, specimen bubbling, specimen handling, and *I*_c_ repeatability.
- The results of this comparison indicate that a more restrictive technique than that which was used by the participating laboratories may not be necessary, and a consensus standard for *I*_c_ measurements could be developed.
- This interlaboratory comparison indicates a level of maturity that this technology has reached.

## Figures and Tables

**Table 1:** Specimen class definitions

Class	Premeasured	Premounted (substrate material)	Preinstrumented	Routing
A	No	No	No	Parallel
B	Yes	No	Partial^a^	Parallel
C	Yes	G-10	Yes	Parallel
D	Yes	G-10	Yes	Series
E	Yes	Brass	Yes	Series
F	Yes	Brass	Yes	Parallel

a These specimens had pressure current contacts, soldered voltage taps, and were measured on G-10 but were not bonded to it.

**Table 2:** Summary statistics of *I*_c_ at 1 μV/cm based on initial NIST zero applied magnetic field measurements for Samples X and Y

	77 K		4.2 K	
Parameter	Sample X	Sample Y	Sample X	Sample Y
*I*_c_ min (A)	18.36	12.14	88.50	79.62
*I*_c_ max. (A)	20.02	16.94	95.11	106.1
*I*_c_ range (%)	8.63	34.42	7.24	29.36
*I*_c_ std. dev. (A)	0.48	1.20	1.80	6.33
*I*_c_ average (A)	19.28	13.95	91.37	90.12
*I*_c_ coef. of var. (%)	2.46	8.60	1.97	7.03
No. of specimens	24	23	24	23
*n*-value average	29.6	28.5	36.2	28.9

**Table 3:** Coefficient of variation (COV) of NIST *I*_c_ data versus magnetic field for 12 specimens at 1 μV/cm

	77 K			4.2 K	
Magnetic field (T)	COV of X (%)	COV of Y (%)	Magnetic field (T)	COV of X (%)	COV of Y (%)
0	2.6	13.1	0	1.7	10.1
0.01	2.9	15.5	0.1	2.2	12.6
0.1	2.6	19.7	1	2.2	13.2
1	4.0	32.0	8	2.5	13.0

**Table 4:** Summary statistics for *I*_c_ normalized to the initial NIST values for the preliminary stage at zero applied magnetic field (Class C and F, Samples X and Y)

Normalized *I*_c_ at 1 μV/cm	77 K		4.2 K	
	Five labs	NIST remeasure	Five labs	NIST remeasure
No. of specimens	19	16	19	16
Average (%)	101.1	99.1	98.3	98.9
Range (%)	7.53	5.20	10.67	3.30
Coef. of var. (%)	2.26	1.16	3.03	1.09

**Table 5:** Summary statistics for *I*_c_ normalized to the initial NIST values for the main stage at zero applied magnetic field (Class B and C, Samples X and Y)

Normalized *I*_c_ at 1 μV/cm	77 K		4.2 K	
	Five labs	NIST remeasure	Five labs	NIST remeasure
No. of specimens	15	16	15	15
Average (%)	98.8	97.3	99.0	97.1
Range (%)	18.24	13.38	11.06	7.43
Coef. of var. (%)	4.36	3.24	3.24	2.76

**Table 6:** Summary statistics for *I*_c_ normalized to the initial NIST values at 77 K versus magnetic field (Class B and C, Samples X and Y)

Normalized *I*_c_ at 1 μV/cm	0.1 T		1.0 T	
	Five labs	NIST remeasure	Five labs	NIST remeasure
No. of specimens	7	16	4	16
Average (%)	181.6	97.6	291.6	98.4
Range (%)	71.99	9.76	160.74	12.51
Coef. of var. (%)	28.76	2.63	74.10	3.20

**Table 7:** Summary statistics for *I*_c_ normalized to the initial NIST values at 4.2 K versus magnetic field (Class B and C, Samples X and Y)

Normalized *I*_c_ at 1 μV/cm	0.5 T		2 T		8 T	
	Five labs	NIST remeasure	Five labs	NIST remeasure	Five labs	NIST remeasure
No. of specimens	16	15	16	15	11	15
Average (%)	96.5	96.9	96.7	97.0	99.9	96.4
Range (%)	16.62	7.74	23.34	7.51	13.12	10.44
Coef. of var. (%)	5.69	2.74	6.75	2.62	4.51	3.61

**Table 8:** Summary statistics of *I*_c_ data at zero applied magnetic field and 1 μV/cm from five laboratories: Class A and B specimens

	77 K		4.2 K	
Parameter	Sample X	Sample Y	Sample X	Sample Y
*I*_c_ min (A)	17.50	11.40	85.00	76.30
*I*_c_ max. (A)	21.01	16.29	96.44	99.47
*I*_c_ range (%)	18.49	37.73	12.69	27.03
*I*_c_ std. dev. (A)	1.11	1.45	3.36	8.24
*I*_c_ average (A)	18.98	12.96	90.16	85.73
*I*_c_ coef. of var. (%)	5.84	11.21	3.72	9.61
No. of specimens	9	9	9	9

**Table 9:** Summary statistics for *I*_c_ normalized to the initial NIST values at 10 μV/cm (Class C and F, Samples X and Y)

Normalized *I*_c_ at 10 μV/cm	77 K		4.2 K	
	Five labs	NIST remeasure	Five labs	NIST remeasure
No. of specimens	18	16	17	16
Average (%)	101.1	99.3	102.2	99.1
Range (%)	7.65	4.78	11.27	3.28
Coef. of var. (%)	2.24	1.02	2.95	0.85

**Table 10:** Laboratory measurement details

Lab	Cooling time room temp. to 77 K (min)	Precool in 77 K	Cooling time 77 K to 4.2 K (min)	Warm between 77 K and 4.2 K	No. *I*_c_ determinations	Specimen cover	Exposed to air below dew point
1	0.5	yes	10	6 no 8 yes	1	no	yes
2	15 – 20	no	N/A	yes	1 or 2	yes	yes
3	25	yes	5	no	1	yes	yes
4	10	some	10	no	1	yes	yes
5	5	yes	3	no	1	no	yes
NIST	5 – 10	no	10 – 15	yes	3	yes	no

**Table 11:** Laboratory mounting details

Lab	Composition of solder by mass fraction (%)	Melting point of solder	Iron temperature	Class A and B^a^ mounting details
1	In: 66	78 °C	100 °C	G-10 substrate, not bonded to substrate, *I* and
	Bi: 34			*V* leads were soldered, specimen held by current leads that were soldered to G-10
2	Pb	unknown	371 °C	G-10 substrate, Nb-Ti current leads were
	Sn			clamped to the G-10 and hold specimen down
3	Sn: 50	145 °C	260 °C to	Specimen was held to substrate by soldered
	Pb: 32	300 °C		current contacts
	Cd: 18			
4	In: 100	157 °C	unknown	G-10 substrate, specimen bonded to substrate
5	In: 98	148 °C	160 °C	G-10 substrate, soldered current leads held specimen down
	Ag: 2			
NIST^b^	In: 52	118 °C	180 °C	G-10 substrate, not bonded to substrate,
	Sn: 48			pressure current leads and soldered voltage leads, specimen was held by the pressure contacts

a Class B specimens had soldered voltage leads.

b Mounting details are for Class B specimens only.
